# Non-adherence to anti-tuberculosis treatment among internal migrants with pulmonary tuberculosis in Shenzhen, China: a cross-sectional study

**DOI:** 10.1186/s12889-015-1789-z

**Published:** 2015-05-08

**Authors:** Ying Tang, Meigui Zhao, Yunxia Wang, Yanhong Gong, Xiaoxv Yin, Angui Zhao, Juanjuan Zheng, Zhenyang Liu, Xiaofang Jian, Wenxin Wang, Chunmei Wu, Zuxun Lu

**Affiliations:** Department of Tuberculosis Prevention and Cure, Bao’an District Hospital for Chronic Diseases Prevention and Cure, Shenzhen, Guangdong 518101 People’s Republic of China; Bao’an District Key Medical Department of Tuberculosis prevention and control, Bao’an District Hospital for Chronic Diseases Prevention and Cure, Shenzhen, Guangdong 518101 People’s Republic of China; Department of Social Medicine and Health Management, School of Public Health, Tongji Medical College, Huazhong University of Science and Technology, Wuhan, Hubei 430030 People’s Republic of China; Department of Public Utilities Management, School of Management, Jiangsu University, Zhenjiang, Jiangsu 212013 People’s Republic of China

**Keywords:** Tuberculosis, Compliance, Migrants

## Abstract

**Background:**

Non-adherence to tuberculosis (TB) treatment threatens the success of treatment, increases the risk of TB spread, and leads to the development of drug resistance. The present study assessed non-adherence to anti-TB treatment among internal migrants with pulmonary TB living in Shenzhen, China, and examined risk factors for non-adherence in order to identify targets for intervention.

**Methods:**

A total of 794 internal migrants with TB treated at Bao’an Hospital for Chronic Disease Prevention and Cure, Shenzhen, were recruited. Structured questionnaires were used to collect data on these patients’ history and experiences with TB treatment. Ordinal logistic regression model were used to identify risk factors for non-adherence.

**Results:**

The proportion of patients who had missed one dose of medication within two weeks was 93/794 (11.71%), and those who missed at least two doses of medication within two weeks was 167/794 (21.03%), with a total of 33.74% of patients not adhering to TB treatment. Lack of knowledge about TB treatment and longer travel time to the nearest community health centers are significant predictors for non-adherence.

**Conclusions:**

The present study shows that non-adherence is common among internal migrants with TB. Patients who lack knowledge about TB treatment or have to travel far to get treated are prone to miss one or more doses of medication. Interventions to improve health education and healthcare access are essential to reduce non-adherence to TB treatment among internal migrants.

## Background

Tuberculosis (TB) remains a major global health problem. According to the World Health Organization (WHO), there were 8.6 million new cases of TB and 1.3 million died from this disease worldwide in 2012 [1]. China accounted for 12% of total TB cases worldwide [1]. Data from the Fifth National TB Epidemiological Survey in 2010 showed that there were about 4.99 million TB patients in China, with a prevalence rate of active pulmonary TB of 459/100,000, and a prevalence rate of sputum smear positive TB of 66/100,000 [2].

Besides active surveillance and prevention, treatment adherence is of the greatest importance in TB control. A standard anti-TB treatment requires the use of a number of medications regularly for a period of at least 6 months for a new patient and 8 months for a retreatment patient, which has been implemented in programs such as Directly Observed Therapy, Short-course (DOTS), promoted vigorously by WHO [3]. Many factors from patient activation to care access can come into play and undermine the treatment adherence, threatening the success of treatment, increasing morbidity and mortality, causing drug resistance, and increasing the risk of TB spread [4].

TB treatment adherence is especially problematic among China’s internal migrants, those who live or work outside their counties of origin for extended periods. Previous studies suggest that internal migrants suffer a disproportionate burden of TB in China [5,6]. The proportion of cases cured among permanent residents was 90.6%, compared with only 37.0% of cases among internal migrants [5]. These internal migrants, now numbering about 236 million nationwide, are mostly young adults who have relocated from poor rural areas to seek a better livelihood in cities [7]; they tend to live and work in crowded environments, far from their families and communities, poor, with limited education, and are less likely to seek medical care when they become ill [8]. For these reasons, internal migrants face much greater challenges in TB treatment adherence than permanent residents do.

This study intended to explore non-adherence in TB treatment among internal migrants with TB in the city of Shenzhen. A highly mobile, dynamic city that spearheaded China’s market capitalism reform, Shenzhen has seen its population growing from 12 thousand in 1980 to 7.7 million in 2012 [9], with internal migrants accounting for more than 70% of the population [9]. By interviewing a sample of TB patients living in Bao’an District, the largest district of Shenzhen, we examined risk factors for non-adherence to TB treatment in order to identify targets for intervention.

## Methods

The study protocol was approved by the Research Ethics Committee of Huazhong University of Science and Technology, Wuhan, China. All the participants read the purpose statement of the investigation and each provided a written informed consent.

### Setting and study population

Tuberculosis services in Bao’an District are provided through a vertical system consisting of the TB departments at the Bao’an District Hospital for Chronic Diseases Prevention and Cure (BHCDPC), subdistrict institutions for disease prevention and healthcare, and community health centers (CHCs). Central to China’s TB control endeavor, all health care providers, including public health facilities, hospitals and clinics, are required to refer suspected TB cases to BHCDPC for TB diagnosis and treatment. Subdistrict institutions and CHCs collaborate with BHCDPC to manage patients and ensure patients adherence. All confirmed TB cases are required to undergo a standard anti-TB treatment. Anti-TB fix-dose combination (FDC) products are provided to TB patients free of charge. During the 2-month intensive phase and the subsequent continuation phase of treatment, all TB patients are required to visit the CHCs every day to take medicines. Doctors at the CHCs are responsible for asking patients for their adverse drug reactions and contacting patients regularly to ensure they complete their treatment.

This cross-sectional study was conducted at BHCDPC, the only authorized institution providing TB diagnosis and treatment services for TB patients in Bao’an District. The participants of this study met the following criteria: 1) active pulmonary TB patients registered at BHCDPC and visited it between December 2013 and March 2014; 2) patients who were taking anti-TB medications and had taken them for over half a month; 3) migrant population, i.e. those who had resided in Shenzhen for >3 months but whose *hukou* (household registration) were still held in their homelands but not in Shenzhen; and 4) patients who were willing to participant in the study. During this period, 794 migrant TB patients were enrolled and completed a structural self-administered questionnaire.

### Assessment of TB treatment Non-adherence

The questionnaire was developed based on a review of previous literature and was tested in a pilot study with 15 TB patients to ensure that the questions were clear and understandable to all participants. The questionnaire included five sections: socio-demographic characteristics (gender, age, educational level, marital status, and employment status); social support; knowledge and attitude towards anti-TB treatment; travel time from home to the nearest CHC; and adherence to anti-TB treatment. In addition, patients’ clinical characteristics including the history of prior anti-TB treatment and sputum smear status were extracted from China’s National TB Information Management System (TBIMS), an internet-based TB case report and management system.

We used an open-ended question to define non-adherence to TB treatment: “Over the past two weeks, how many doses of anti-TB medication did you miss?” Patients who had missed at least one prescribed dose of TB drug were deemed as non-adherence.

Social support for patient was assessed by the Social Support Rating Scale (SSRS) [10], which has been widely used in studies of Chinese populations, demonstrating high reliability and validity and high internal consistency [11,12]. The SSRS has ten items to evaluate total social support, including three items for levels of objective support, four items for subjective support, and three items for the utility of support. The total score is used as a measure of the current total social support status, ranging from 12 (worst possible social support) to 66 (best possible social support). The score was divided into three categories based on its mean and standard deviation in our analysis.

Patients’ knowledge and attitude towards anti-TB treatment was measured by three questions: 1) “Can TB patients stop anti-TB treatment by themselves when mild adverse drug reactions appear?” 2) “It does not matter that a TB patients misses some doses of anti-TB medication, doesn’t it?” and 3) “Does a TB patient need to keep anti-TB treatment up when symptoms disappear?” All these questions have three optional answers: yes, no or do not know.

### Statistical analysis

The data were double-entered into EpiData 3.0 for Windows (EpiData Association, Odense, Denmark) by two individuals separately. All statistical procedures were performed using the Statistical Analysis System (SAS) 9.2 for Windows (SAS Institute Inc., Cary, NC, USA). Rank-sum tests were conducted to compare missing doses of anti-TB medication within two weeks across groups defined by socio-demographic data, social support, travel time to the nearest CHC, clinical characteristics, and knowledge and attitude towards anti-TB treatment. Ordinal logistic regression analysis was used to analyze the potential risk factors of non-adherence. All comparisons were two-tailed. The significance threshold was set at p = 0.05.

## Results

Table 1 presents the rates of non-adherence to TB treatment by participants characteristics. The age of the patients ranged from 14 to 84 years old, with mean age of 32.57 (standard deviation (SD): 10.93) years. Overall, 33.74% of the patients were considered non-adherent. The proportion of patients who had missed one dose and more than one dose of medication within two weeks were 11.71% and 21.03%. Patients with lower education level, single, employed, newly diagnosed, living farther away from CHC had higher rates of non-adherence, and their lack of knowledge about anti-TB treatment was also associated with higher non-adherence rates. Table 2 shows that, among the reasons queried, forgetfulness (64.62%), too busy (69%), and running out of anti-TB pills (41.54%), were the most frequently cited for missing the anti-TB pills (Table 2).

**Table 1:** **Participant characteristics and their associations with missing doses of anti-TB medication within two weeks**

		**Missed doses in two weeks (%)**			
**Variables**	**n (%)**	**0**	**1**	**2+**	***P***
**Total**	794	67.25	11.71	21.03	
**Age**					0.47
<25	202 (25.44)	65.35	10.40	24.26	
25~	318 (40.05)	65.09	14.78	20.13	
35~	164 (20.65)	73.17	8.54	18.29	
45~	67 (8.44)	71.64	5.97	22.39	
55~	43 (5.42)	62.79	16.28	20.93	
**Gender**					0.97
Female	284 (35.77)	66.90	12.68	20.42	
Male	510 (64.23)	67.45	11.18	21.37	
**Educational level**					**0.02**
Primary school or less	69 (8.69)	72.46	11.59	15.94	
Junior high school	378 (47.61)	71.16	10.85	17.99	
Senior high school	232 (29.22)	59.91	12.07	28.02	
Junior college or above	115 (14.48)	66.09	13.91	20.00	
**Marital status**					**0.04**
Married/cohabitation	483 (60.83)	69.57	12.42	18.01	
Single/widow/divorced	311 (39.17)	63.67	10.61	25.72	
**Employment status**					**0.02**
Employed	580 (73.05)	65.00	12.24	22.76	
Unemployed	214 (26.95)	73.36	10.28	16.36	
**Social support**					0.06
Good	135 (17.00)	68.89	11.85	19.26	
Fair	530 (66.75)	65.09	11.32	23.58	
Poor	129 (16.25)	74.42	13.18	12.40	
**History of prior anti-TB treatment**					0.06
New case	718 (90.43)	66.30	11.84	21.87	
Retreated case	76 (9.57)	76.32	10.53	13.16	
**Sputum smear status**					0.15
Positive	449 (56.55)	69.27	11.36	19.38	
Negative	345 (43.45)	64.64	12.17	23.19	
**Travel time to the nearest CHC**					**0.04**
<15 Mins	456 (57.43)	69.52	13.38	17.11	
≥15 Mins	338 (42.57)	64.20	9.47	26.33	
**Can TB patients stop anti-TB treatment by themselves when mild adverse drug reaction appears?**					**0.0013**
No, they cannot.	412 (51.89)	71.84	13.59	14.56	
Yes, they can.	170 (24.41)	59.41	11.76	28.82	
Not sure	212 (26.70)	64.62	8.02	27.36	
**It does not matter that TB patients miss some doses of anti-TB medication, does it?**					**0.0027**
Yes, it does.	652 (82.12)	69.79	11.35	18.87	
No, it does not.	48 (6.05)	56.25	16.67	27.08	
Unknown.	94 (11.84)	55.32	11.70	32.98	
**Does TB patient need to keep anti-TB treatment up when symptoms disappear?**					0.52
Yes.	438 (55.16)	68.26	12.10	19.63	
No.	95 (11.96)	67.37	16.84	15.79	
Unknown.	261 (32.87)	65.52	9.20	25.29	

Note: *P* values are associated with rank-sum tests.

**Table 2:** **Reasons of missing doses of anti-TB medication**

	**Missing doses in two weeks**				**Total**	
	**1 (N = 93)**		**2 + (N = 167)**			
	**n**	**%**	**n**	**%**	**n**	**%**
To avoid side effects.	2	2.15	9	5.39	11	4.23
Too busy.	41	44.09	109	65.27	150	57.69
Forgetfulness.	44	47.31	124	74.25	168	64.62
Anti-TB pills have been run out.	32	34.41	76	45.51	108	41.54
Worrying that somebody may despise me.	2	2.15	4	2.4	6	2.31
Confusing about the dose of anti-TB medicines.	0	0	1	0.6	1	0.38
Travelling to other places.	14	15.05	32	19.16	46	17.69
Others	6	6.45	7	4.19	13	5

Table 3 presents the results from the ordinal logistic regression model. Controlling for confounding, TB patients who needed 15 minutes or more time to travel from home to the nearest CHC were more likely to miss doses of anti-TB medication than those who took less than 15 minutes (OR = 1.41). Participants who believed or were not sure that TB patients could stop anti-TB treatment by themselves when mild adverse drug reactions appear were more likely to miss doses of medication than those who believed otherwise (OR = 1.58). The respondents who did not consider or were not sure that it did matter that TB patients miss some doses of anti-TB medication had higher odds for missing doses of medication (OR = 1.89).

**Table 3:** **Ordinal logistic regression analysis of missing any dose of anti-TB medication in two weeks with mutually adjusted ORs and 95% CI**

	**OR (95% CI)**	***P***
**Age (Ref** ^**§**^ **: <25)**		
25~	1.12 (0.74-1.69)	0.59
35~	0.94 (0.55-1.59)	0.81
45~	1.12 (0.57-2.21)	0.75
55~	1.50 (0.70-3.21)	0.30
**Gender** **(Ref: Female)**		
Male	0.92 (0.66-1.28)	0.61
**Educational level** **(Ref: Primary school or less)**		
Junior high school	1.10 (0.60-2.02)	0.75
Senior high school	1.77 (0.93-3.36)	0.08
Junior college or above	1.28 (0.62-2.62)	0.50
**Marital status** **(Ref: Married/cohabitation)**		
Single/widow/divorced	1.24 (0.85-1.80)	0.27
**Employment status** **(Ref: Employed)**		
Unemployed	0.68 (0.47-0.98)	**0.04**
**Social support** **(Ref: good)**		
Fair	1.09 (0.71-1.67)	0.68
Poor	0.66 (0.37-1.17)	0.15
**History of prior anti-TB treatment** **(Ref: New case)**		
Retreated case	0.61 (0.34-1.09)	0.09
**Sputum smear status** **(Ref: Positive)**		
Negative	1.09 (0.80-1.49)	0.59
**Travel time to the nearest CHC** **(Ref: <15Mins)**		
≥15 Mins	1.41 (1.04-1.92)	**0.03**
**Can TB patients stop anti-TB treatment by themselves when mild adverse drug reaction appears?** **(Ref: No, they cannot.)**		
Yes, they can./Not sure.	1.58 (1.16-2.15)	**<0.01**
**It does not matter that TB patients miss some doses of anti-TB medication, does it?** **(Ref: Yes, it does.)**		
No, it does not./Unknown.	1.89 (1.30-2.74)	**<0.01**
**Does TB patient need to keep anti-TB treatment up when symptoms disappear?** **(Ref: Yes.)**		
No./Unknown.	0.99 (0.73-1.35)	0.96

^§^Ref is reference.

## Discussion

The present study demonstrated that non-adherence was common among internal migrants in China. Some previous studies in China also indicated low treatment adherence among TB patients [13,14]. A study recruited 524 TB patients in mountain areas indicated that 9.4% of patients took less than 90% of all doses prescribed [15]. Hu et al. reported that 12.5% of patients had not taken any TB drugs in the previous week [14]. Another study conducted in Jiangsu Province in China reported that 12.2% of patients missed at least 10% of their prescribed doses of anti-TB medication [16]. A recent study in Shandong Province in China found that 16% of migrant TB patients did not adherent to TB therapy [13]. The proportion of non-adherence in our study is higher than that were reported in previous studies, and much of the difference may be due to differences in study design and definition of non-adherence to anti-TB treatment. In our study, we employed TB patient’s self-reported drug-taking history within two weeks before the interview, which could have in some extent reduced recall bias. Another frequently used definition of non-adherence is the WHO recommended measure, which defines a TB treatment defaulter as a patient who interrupted treatment for two consecutive months or more [17]. Furthermore, a few studies considered a patient who had missed 10% or more of the total prescribed doses of TB drugs as non-adherent [15,16]. Regardless, our estimate is consistent with previous studies to indicate that non-adherence is a serious problem among migrant TB patients.

Our study shows that, among the migrant TB patients, lack of knowledge about TB treatment and longer travel time to the nearest CHC were the most significant factors predicting non-adherence, consistent with earlier studies. Driver et al. explored the determinants for treatment interruption and indicated that lack of TB and its treatment related knowledge were important reasons for discontinuing treatment [18]. Many studies have illustrated a positive effect of health education on improving treatment adherence. A cluster randomized trial in Senegal showed that intensive strategy of treatment monitoring and education led to improved adherence to medications and improved outcomes among TB patients [19]. In fact, the National TB Control Program Implementation Guide in China (2008 Edition) states clearly that carrying out health education for TB patients before chemotherapy was the most important step of DOTS implementation, and the treatment regimen and importance of adherence were the core of health education. In our study site, doctors implement health education for all TB patients before chemotherapy. However, some participants were still confused about treatment regimen after health education, indicating the need for improvement in the health education segment of the program.

Our study indicated that, after controlling for patient knowledge on TB treatment and travel time to the nearest CHCs, non-adherence to anti-TB treatment was not associated with migrant TB patients’ socio-demographic characteristics such as gender, age, educational level, marital status and employment status. The associations between non-adherence and demographic characteristics have been explored in many studies and their results are inconsistent [20–22]. For example, marital status is reported to be associated with adherence to anti-TB treatment [13]; some researchers believed that family members, especially the spouse, played an important role in treatment supervision. However, participants in our study needed to take anti-TB medication in healthcare facilities every day, and the supervision of their family members was not indispensable. Furthermore, of the internal migrants, many married people lived separately from their spouses. Another of our findings that seem inconsistent with previous studies is on the association between social support and adherence [22]. It is worth noting that social support is relatively poor in all migrant TB patients, and our social support measure using SSRS might not have meaningfully distinguish those with strong versus weak social support.

Several limitations of this study need to be stated. Firstly, the treatment adherence levels were based on self-reported information from patients, so recall bias was unavoidable. Secondly, the occurrence of adverse drug reactions of taking anti-TB medications, which is a determinant of the non-adherence, was not taken into consideration in our analysis.

## Conclusion

Our study indicates that non-adherence among migrant pulmonary TB patients is common and patients’ knowledge about anti-TB treatment is crucial to improve migrant pulmonary TB patients’ treatment adherence. In the TB treatment program, health care providers should assure that all patients understand the core knowledge of anti-TB treatment regimen and the importance of adherence. In addition, efforts in improving the migrant TB patients’ general access to care may also help them to complete TB treatment.

## Abbreviations

TB: Tuberculosis
WHO: World Health Organization
DOTS: Directly observed therapy, short-course
BHCDPC: Bao’an district hospital for chronic diseases prevention and cure
CHCs: Community health centers
SSRS: Social support rating scale
OR: Odds ratio
